# The Influence of the COVID-19 Pandemic on the Sexual Lives of Polish Young Adults

**DOI:** 10.3390/jcm13123370

**Published:** 2024-06-07

**Authors:** Helena Perenc, Karolina Pasieka, Kamil Juruś, Karolina Bierć, Rafał Bieś, Marek Krzystanek, Anna Warchala

**Affiliations:** 1 Students’ Scientific Association, Department and Clinic of Psychiatric Rehabilitation, Faculty of Medical Sciences in Katowice, Medical University of Katowice, ul. Ziołowa 45/47, 40-635 Katowice, Poland; perenc.helena@gmail.com (H.P.); pasieka.karolina3@gmail.com (K.P.); kamiljurus360@gmail.com (K.J.); bierckarolina@gmail.com (K.B.); s82700@365.sum.edu.pl (R.B.); 2 Department and Clinic of Psychiatric Rehabilitation, Faculty of Medical Sciences, Medical University of Silesia in Katowice, ul. Ziołowa 45/47, 40-635 Katowice, Poland; m.krzystanek@sum.edu.pl

**Keywords:** COVID-19 pandemic, sexual health, lockdown measures, sexual dysfunction

## Abstract

**Background**: The COVID-19 pandemic brought significant changes to daily life in Poland, with restrictions affecting various sectors, including entertainment, education, and travel. The pandemic’s impact extended to intimate aspects of life. This study aimed to compare the sexual functioning of young adults in Poland before and during the pandemic, using the Changes in Sexual Functioning Questionnaire (CSFQ-14). **Methods**: The research involved an online survey with demographic questions, the CSFQ-14 for pre-pandemic sexual functioning, and modified CSFQ-14 questions for the pandemic period. Sexual dysfunction was determined using predefined cutoff scores. **Results**: Overall, the study found no significant difference in the sexual functioning of young Poles during the COVID-19 pandemic compared to before. However, there were gender-specific trends. Women reported enhanced pleasure, satisfaction, and orgasm during lockdown, while men faced challenges with erection and ejaculation. A higher proportion of women experienced overall sexual dysfunction compared to men, both before and during the pandemic. This research provides insights into the impact of the COVID-19 pandemic on the sexual lives of young Poles. While overall sexual functioning remained relatively stable, specific aspects varied by gender. **Conclusions**: The study emphasizes the need to consider demographic factors, such as age and gender, when assessing the effects of external stressors like a pandemic on sexual health. Further research is essential to fully grasp these complexities and their potential long-term consequences.

## 1. Introduction

Coronavirus disease 2019 (COVID-19) is an infectious disease caused by severe acute respiratory syndrome coronavirus 2 (SARS-CoV-2). The first known case was identified in the city of Wuhan, located in China, in December 2019. Symptoms of COVID-19 include cough, fever, fatigue, sore throat, shortness of breath, and myalgia [1,2,3]. The first COVID-19 case in Poland was confirmed on 4 March 2020 in Zielona Góra [4]. From 14 to 20 March 2020, an epidemic emergency was introduced [5]. On 20 March 2020, an epidemic state was introduced [6]. On 3 March 2023, the Polish government reported a total of 6,437,598 confirmed cases, of which 5,335,807 people recovered and 118,970 died [7]. 

During the COVID-19 pandemic, various restrictions were introduced to protect Polish citizens—there were periods of strict lockdowns, as well as the relaxation of no-contact policies. Nevertheless, the pandemic affected people significantly, both in Poland and around the world. As Tan et al. and Toldam et al. underline, its impact on life was complex and modified by numerous factors [8,9]. The pandemic exacerbated mental health issues, resulting in increased levels of depression and anxiety, which further deteriorate overall quality of life. Moreover, the disease affected various aspects of daily functioning, including mobility, cognitive function, and participation in life activities, with post-COVID symptoms exacerbating these issues [10].

Sexual well-being is an integral part not only of everyday life, but also of general health [11]. The WHO acknowledge the importance of sexual health and define it as a “state of physical, emotional, mental and social well-being in relation to sexuality; it is not merely the absence of disease, dysfunction or infirmity” [12]. Good sexual functioning is connected to better quality of life and overall higher life satisfaction in various socio-demographic groups in different countries [12,13,14,15,16,17,18,19]. Sexual dysfunctions, however, are a heterogenous group of disorders that typically involve an impairment of a person’s ability to respond sexually or a disturbance in the feeling of sexual pleasure [20].

Thus, given the importance of sexual functioning, it is vital to examine the impact of the COVID-19 pandemic on the sexual lives of individuals. Numerous studies have been conducted to assess changes in sexual behaviors during this world health crisis. The high prevalence of anxiety and depression during the lockdown period influenced sexual functioning negatively [9,21,22,23,24,25]. Sexual behavior was also impacted by the perception of COVID-19 as a health risk, as well as the fear of transmission of the virus via sexual contact [22,23,26,27,28,29]. It is worth mentioning that the effects of the infection itself can be detrimental to sexual health—it has been described that COVID-19 may lead to issues regarding genitourinary function, both in male and female patients [30,31,32,33,34,35]. Moreover, sexual, and reproductive health services were less available during the lockdown period [23,36]. Changes in sexual behaviors were observed by many researchers. For example, masturbation [36,37,38,39,40], the use of technologies to arrange virtual dates [39], and watching pornography [25,37,38,41,42,43] became more frequent.

According to González-Sanguino, who performed the first study regarding the psychological effects of the COVID-19 outbreak in the population of Spain, older age served as a protective factor against developing symptoms of depression, anxiety, and PTSD during the lockdowns [44]. This suggested that younger people may have been more susceptible to the adverse effects of the pandemic on their mental health. These findings highlighted the importance of addressing the impact of the pandemic on the well-being of young adults, including sexual functioning. 

Due to rising population density, continued deforestation, closer human–animal contact, and increased rapid international travel, the occurrence of future epidemics and pandemics seems inevitable [45]. The results of this study may therefore contribute to a better understanding of sexual dysfunctions, which in turn could be helpful in the development of prevention methods for these disorders in the future. 

The aims of this study were to compare the sexual functioning of the young Polish population during the COVID-19 pandemic to the period before the pandemic and to identify the factors which differentiated the sexual functioning of young Polish people before the COVID-19 pandemic and during the pandemic. To the best of our knowledge, no prior research has been published on the impact of the COVID-19 pandemic on the sexual life of Poles using the Changes in Sexual Functioning Questionnaire (CSFQ-14). This is the first study of its kind, which may provide a new perspective on the issue under investigation.

## 2. Materials and Methods

### 2.1. Research Instruments

The study was conducted with an online questionnaire divided into three parts:

1. Questions about sex, age, place of residence, religiosity, relationship status, quarantine or self-isolation during the lockdown period, remote work, and remote university studies.
2. Changes in Sexual Functioning Questionnaire—CSFQ-14: a 14-item scale [46,47,48] in relation to the pre-pandemic period.
3. CSFQ-14 questions modified in relation to the pandemic period.

The CSFQ was developed in 1997 and has been proven to be a useful, reliable, and valid measure of sexual function for a research setting [46]. The CSFQ-14 version was used and its scores were classified according to its design. The CSFQ-14 is available in two versions—female and male—both of which contain five subscales: pleasure, desire/frequency, desire/interest, arousal/excitement and orgasm/completion for females, and pleasure, desire/frequency, desire/interest, arousal/erection and orgasm/ejaculation for males. The range of total CSFQ-14 scores is 14–70 points, with cut-off points of sexual dysfunction at 41 points or lower for females and 47 or lower for males [46,47,48].

### 2.2. Time of Conducting the Research

The study was conducted between 5 March 2021 and 1 April 2021. In this research, the pandemic period was defined as the time since the beginning of the COVID-19 pandemic in Poland (March 2020) to the moment of participation in the study. To simplify, it was the first year of the COVID-19 pandemic in Poland, which consisted of various periods of different levels of restrictions: full lockdown, relaxations, and further lockdowns (depending on the epidemiological situation), but mostly periods of tight restrictions, e.g., online schooling, recommendations of remote work, and an unavailability of public places except for those designated to fulfill basic needs. Sexual functioning in the pre-pandemic period was defined as usual sexual functioning before the beginning of the COVID-19 pandemic in March 2020.

### 2.3. Study Sample

The subjects were selected randomly. The survey was conducted online using random sampling selection. Communication via social media, such as Facebook or Instagram, was used to enroll participants from different subgroups differentiated by sex, age, place of residence, religiosity, relationship status, quarantine or self-isolation during the lockdown period, remote work, and remote university studies.

### 2.4. Inclusion Criteria

The target population was young adults, aged 18–27, living in Poland. Participation in the study was voluntary. To be included in the analysis, participants were required to complete the whole questionnaire. The participants were informed that the data would be used for research purposes, and they agreed to it by completing the survey.

### 2.5. Study Group Characteristics

The study covered a population of 541 people. The study group characteristics are shown in Table 1. 

### 2.6. Statistical Analysis

The Wilcoxon test was used to compare CSFQ-14 scores of individuals referring to the periods before and during the pandemic. The Mann–Whitney test and the Kruskal–Wallis ANOVA test were used to compare CSFQ-14 scores in subgroups divided by specific features. The Chi2 test was used to compare the percentages of participants with CSFQ-14 scores indicative of normal functioning or dysfunction in subgroups divided by different factors. The Shapiro–Wilk test was performed to assess the distribution of CSFQ-14 scores. A *p* value lower than 0.05 was defined to be indicative of statistical significance. To examine the compliance of CSFQ-14 scores with normal distribution, the Shapiro–Wilk test and visual histogram analysis were used. The scores did not follow a normal distribution. Statistica TIBCO 13.3 was used.

### 2.7. Ethical Aspects

The study was approved to be conducted by the Bioethics Committee of the Silesian Medical University in Katowice (No. PCN/0022/KB/13/21, 29 January 2021).

## 3. Results

### 3.1. Total CSFQ-14 Scores

Table 2 shows the total CSFQ-14 scores of both groups (male and female) describing overall sexual functioning before and during the COVID-19 pandemic. There was no statistically significant difference between the CSFQ-14 scores of individuals before and during the pandemic (Wilcoxon test, *p* > 0.05).

In men, both before and during the pandemic, place of residence, religiosity, relationship status, sexual orientation, cohabitation with a partner, remote work, remote university studies, or an episode of quarantine or self-isolation during the pandemic did not differentiate the total CSFQ-14 score (*p* > 0.05). In every subgroup divided by these factors, the median of the total CSFQ-14 score was indicative of normal sexual function. 

In women, both before and during the pandemic, place of residence, religiosity, relationship status, sexual orientation, cohabitation with a partner, remote university studies, or an episode of quarantine or self-isolation during the pandemic did not differentiate the total CSFQ-14 score (*p* > 0.05). Remote work did not significantly influence the total CSFQ-14 score before the pandemic; however, in the period of the COVID-19 pandemic, women who worked remotely had a significantly lower score than women who did not work remotely (*p* = 0.004). In every subgroup divided by these factors, the median of the total CSFQ-14 score was indicative of normal sexual function. Table 3 shows the specific scores of the subgroup differentiated by remote work.

### 3.2. Percentage of People with Scores Indicative of Dysfunction

There was no statistically significant difference in the percentage of people whose scores were indicative of normal sexual functioning or dysfunction before and during the COVID-19 pandemic.

There was a significant difference in the percentages of scores indicative of sexual dysfunction in men and women both before the pandemic (*p* = 0.0002) and during the pandemic (*p* = 0.02). Only 1.63% of men obtained scores indicative of sexual dysfunction before the pandemic, whereas in women this value was 14.59%. During the pandemic, only 4.88% of men displayed sexual dysfunction, whereas in women the percentage was 12.20%.

In the subgroups differentiated by place of residence, religiosity, relationship status, sexual orientation, cohabitation with a partner, remote university studies, or an episode of being quarantined or self-isolated because of COVID-19, there were no significant differences in the percentages of total CSFQ-14 scores indicative of sexual dysfunction, both before the pandemic and during the pandemic. This was observed when taking into consideration the study group, as well as women and men separately. Our findings about the differences in the percentages of participants with normal sexual functioning (according to CSFQ-14) in groups of men and women are summarized in Table 4.

Performing remote work did not influence the percentage of scores indicative of dysfunction during the COVID-19 pandemic. However, before the pandemic, in the subgroup who used to work remotely, the percentage of people with sexual dysfunction was significantly lower (*p* = 0.045). This was especially prominent in women. Before the pandemic, only 3.12% of women who worked remotely showed signs of overall sexual dysfunction, whereas in the group of women who did not perform remote work before the COVID-19 pandemic, the percentage was 15.54% (*p* = 0.004). These findings are summarized in Table 5.

### 3.3. CSFQ-14 Subscales

In general, during the COVID-19 pandemic, men were observed to function significantly worse in terms of erection (*p* = 0.003) and ejaculation (*p* = 0.022) than before the pandemic. Women were noted to function significantly better in terms of pleasure (*p* = 0.019) and orgasm (*p* = 0.005) during the pandemic in comparison to the time before the pandemic. In men, the medians of the CSFQ-14 subscales of pleasure, desire/frequency and desire/interest were scores indicative of sexual dysfunction both before and during the pandemic (with cut-off points of ≤4/4, ≤6/8 and ≤9/11, respectively). The median score for the male orgasm scale was indicative of dysfunction in relation to the time during the pandemic (with a cut-off point of ≤11/13). In women, the medians of the CSFQ-14 subscales of pleasure and arousal were scores indicative of sexual dysfunction both before and during the pandemic (with cut-off points of ≤4/4 and ≤12/13, respectively). The median score for the female orgasm scale was indicative of dysfunction in relation to the period before the COVID-19 pandemic (with cut-off point of ≤11/13). These findings are summarized in Table 6.

Regarding the differences between sexual functioning in different time periods, during the COVID-19 pandemic, a significantly lower percentage of women obtained scores indicative of sexual dysfunction in terms of sexual pleasure (cut-off point of ≤4/4 in CSFQ-14-Pleasure) in comparison to the time before the pandemic (*p* < 0.00001). These findings are summarized in Table 7. In terms of other subscales, no differences were found in the percentages of people with sexual dysfunction during the pandemic in comparison to the period before the pandemic, in both the group of men and the group of women.

Regarding the differences between male and female participants, both before and during the COVID-19 pandemic, there were no differences in the percentages of men and women with sexual dysfunction in terms of pleasure and orgasm. Before the COVID-19 pandemic, a higher percentage of men than women obtained scores indicative of sexual dysfunction in terms of desire and frequency (*p* < 0.0001), and a higher percentage of women obtained scores showing sexual dysfunction in terms of sexual arousal (*p* < 0.0001). Before the COVID-19 pandemic, a higher percentage of men also showed scores indicative of sexual dysfunction in terms of desire and interest (*p* = 0.01). These findings are shown in Table 8.

In men, both before and during the pandemic, place of residence, religiosity, relationship status, sexual orientation, cohabitation with a partner, remote work, remote university studies, or an episode of quarantine or self-isolation during the pandemic did not differentiate the sexual functioning reflected by the CSFQ-14 scores of pleasure, desire/frequency, desire/interest, arousal/erection, or orgasm/ejaculation (*p* > 0.05).

In women, both before and during the pandemic, place of residence, religiosity, relationship status, sexual orientation, cohabitation with a partner, remote university studies, or an episode of quarantine or self-isolation during the pandemic did not differentiate the sexual functioning reflected by the CSFQ-14 scores of pleasure, desire/frequency, desire/interest, arousal, or orgasm (*p* > 0.05). Remote work before the COVID-19 pandemic seemed to significantly positively influence the scores of desire/frequency (*p* = 0.01) and orgasm (*p* = 0.03) in women. However, remote work during the pandemic significantly lowered the scores in terms of desire/interest (*p* = 0.01) and arousal (*p* = 0.003) in women. These findings are shown in Table 9.

## 4. Discussion

The coronavirus disease pandemic, according to the results of this study, had some impact on the sexual functioning of young people, but in general, sexual functioning did not significantly differ compared to the period before the pandemic, reflected by the lack of statistically significant differences in total CSFQ-14 results describing the sexual functioning of individuals before and during the lockdown period. Moreover, the medians of the total CSFQ-14 scores for men and women were indicative of normal sexual functioning. Similar findings were described by Arafat et al., who examined the populations of India, Nepal and Bangladesh. Although 45% of their participants reported that the pandemic affected their sexual life, the data showed no significant difference in sexual activity between the time periods before and during the lockdown [49]. The effect of the changes was not major, and the frequency of sexual activity did not change significantly [49]. In a study by Panzeri et al., it was also reported that most Italian couples who had been interviewed did not perceive any difference in their sexual functioning [50]. In the study group of Gauvin et al., only minimal disruptions in sexual functioning were noted and the authors underlined that the changes in sexual functioning and relationships were not significant enough to be considered major health problems [51]. What is more, in the meta-analysis performed by Dashti et al., it was concluded that there was no significant difference in total FSFI score, or its domains, in the female population between the pre-pandemic period and the COVID-19 pandemic period [52].

On the other hand, the findings of some authors directly contradict the results of this study. Omar et al. noted that in both male and female respondents from Egypt, sexual satisfaction was significantly lower during the lockdown period than before [21]. According to Cocci et al., the percentage of people in the Italian study sample who reported lack of sexual satisfaction increased significantly during quarantine (from 7.46% before the pandemic to 53.53% during quarantine, *p* < 0.01) [37]. Karagoz et al. described deteriorated sexual function in comparison to the pre-pandemic period, reflected in significantly lower IIEF-5 and FSFI scores (*p* = 0.001 and *p* = 0.027, respectively) [38]. These tendencies were later confirmed by a meta-analysis by Masoudi et al., who used standardized mean difference to evaluate the results of studies regarding sexual functioning during the COVID-19 pandemic and concluded that the lowering of IIEF-5 and FSFI scores between studies was significant [40]. Many authors underline the adverse effects of the COVID-19 pandemic on the frequency of sexual activities. Cito et al. showed a correlation between sense of well-being and the number of instances of sexual intercourse before and during quarantine and, overall, the number of instances of sexual intercourse decreased significantly during quarantine—mainly because of privacy issues and a lack of psychological stimulation [53]. Karsiyakali et al., Li W. et al., Karagoz et al., Coombe et al., Räuchle, and Baran et al. also point to the significantly lower frequency of sexual activity during the pandemic [26,38,39,42,54,55]. 

The differences between these results and the results of this study may be explained by the characteristics of the study groups. In this study, only young people were examined—the median age was 21 years old. Other authors also describe the significance of age. Batz et al. noted that higher satisfaction with sexual life is correlated with age younger than 36 [56]. Similar findings were described by Lehmiller et al., who describe young age as a factor linked to a higher likelihood of introducing new additions into sexual life, which tends to result in a general improvement of sexual functioning [57]. According to Li W. et al., younger age is also closely related to the frequency of sexual intercourse [55]. Some studies describe tendencies of changes in sexual functioning which seem unclear. According to the study by Ko et al., in the examined Taiwanese population, the frequency of sexual activity and sexual satisfaction improved in 1.6–2.9% of the population, but worsened in 1.4–13% [22]. Lehmiller et al. described the tendencies observed in their online survey in which nearly half of the sample noted a decline in their sexual activity, but also about 20% of participants reported new additions to their sexual activities, such as new sexual positions, sexting, or sharing sexual fantasies, which improved the quality of their sexual life [57]. These findings seem to support the idea that under extraordinary circumstances, such as pandemics, individuals develop different sexual behaviors which may be beneficial, and that young age may be a protective factor from sexual dysfunction. 

This would also explain why, regarding specific aspects of female sexual functioning, the results of this study indicate that during the lockdown period, women tended to function better in the aspects of pleasure/satisfaction and orgasm. These findings are not supported by any other studies. Authors mainly point to a lowered quality of sexual life in women. Cipolletta et al., Schiavi et al., Yuksel and Ozgor, Batz et al., and Omar et al. describe a significant decrease in all aspects of female sexual functioning in different populations [21,56,58,59]. Similar tendencies were noted by Fuchs et al., who studied the changes in sexual functioning of Polish women; significantly decreased FSFI scores in every domain (desire, arousal, lubrication, and pain) were observed, which indicated worse sexual functioning during the COVID-19 pandemic than before (30.1 ± 4.4 vs. 25.8 ± 9.7, *p* < 0.001) [60]. Omar et al. explain that decreased sexual satisfaction during the pandemic may partially be the result of anxiety and depression, which were more prevalent in women [21]. Panzeri et al. also suggest that the reasons for decreased function in women in terms of pleasure, satisfaction, desire, and arousal seem to be psychological distress and a lack of privacy [50]. These findings are contradictory to this study. Unfortunately, in this study, the participants were not asked about mental health, so no clear conclusions about such aspects can be made. However, we can speculate that the unusual tendencies in women in this study may also be a result of the young age of the study population.

Regarding specific aspects of male sexual functioning, the results of this study indicate that during the lockdown period, men tended to function worse in terms of erection and ejaculation. Moreover, Fang et al. described the deterioration of erectile function in Chinese adult males during the pandemic, which was reflected by a significant difference regarding mean IIEF-5 value [61]. Similarly, Szuster et al., who examined Polish males during the pandemic, reported a mean IIEF-15 score in the erectile function domain of 22.27, indicative of mild erectile dysfunction [62]. However, Fang et al. state that the pandemic did not influence the function of ejaculation, as reflected by the mean PEDT scores in their study sample [61]. The results pointing to possible erectile dysfunction in men seem to be consistent; however, in different study groups, the strength of the correlation between lockdown and the deterioration of this specific sexual function seems to vary. The reason for this may be linked to the characteristics of the study groups and different factors which might influence their sexual health. It is possible that the problems with erectile function are due to the increased frequency of masturbation during the COVID-19 pandemic, which, according to Li G. et al., occurred in 30% of respondents [36]. Szuster et al. also underline the fact that during the pandemic, the libido of Polish men decreased significantly [51].

It is worth mentioning that, according to our research, the medians of the total CSFQ-14 scores for men and women were indicative of normal sexual functioning, whereas the specific subscales of CSFQ-14 showed median scores in the dysfunction range regarding the time of the pandemic and before. This suggests that good overall sexual functioning does not exclude the possibility of dysfunction in some aspects of sexual life. In this context, we might even conclude that the data from this study suggesting the unchanged sexual functioning of individuals and the deterioration of specific aspects observed in other studies are not contradictory.

Gender seems to be an important factor which may differentiate sexual functioning. In our study, significantly more women than men suffered from overall sexual dysfunction both before and during the pandemic. Sexual dysfunction in women seems to be directly influenced by psychological factors, which was revealed in a study by Carvalho et al. [63]. Peterson et al. noted that sexual minorities and women generally functioned far less well in the pandemic and experienced higher level of psychological distress [64]. This is also supported by other researchers, such as Omar et al., who noted that during lockdown, significantly more men were satisfied with their sexual performance than women (*p* < 0.001, 70.5% vs. 56.2%, respectively) [23]. The study by Jacob et al. indicates that the number of sexual activities in the lockdown period was significantly higher in men than in women (*p* = 0.002) [65]. Cocci et al. also found that women had greater depression (BDI-male: 8.0 [IQR 4.0–13.0]; BDI-female: 11.0 [IQR 6.0–17.0]; *p* < 0.01) and anxiety levels compared to men (BAI-male: 7.0 [IQR 3.0–14.0]; BAI-female 13.0 [IQR 7.0–23.0]; *p* < 0.01) [37]. In their meta-analysis, Masoudi et al. observed that the adverse effects of the pandemic on sexuality were greater in women in comparison to men [40]. Peyravi et al. stated that during the COVID-19 pandemic, women were considered a sensitive group in need of special care because of numerous challenges at this time, for example, sexual violence and abuse in marriage due to increased conflict during lockdown periods, higher expectations in terms of taking care of children during lockdown, which resulted in less time for self-care, worse access to contraception and healthcare, or fear of COVID-19 transmissions [66]. According to Fuchs et al., the lower FSFI scores were noted in women who did not work during the pandemic; lack of work activity and boredom were considered a risk factor of lowered sexual desire [60]. This would partially explain why, in our study, remote work during the pandemic was associated with a worsening of sexual functioning—similar psychological effects of boredom and routine may be present in the case of working from home without alternative options during lockdown. 

A specific form of work, also associated with stress disorders, which seemed to worsen sexual functioning in different domains during the pandemic was working in healthcare [67,68]. Further research would be needed to establish the role of form of work on sexual functioning and its gender specificity.

It is possible that factors other than gender may influence sexual well-being. According to the results of this study, in women and men, both before and during the pandemic, place of residence, remote university studies, an episode of quarantine or self-isolation during the pandemic, relationship status, or cohabitation with a partner did not impact sexual functioning significantly. Many studies, however, describe the influence of relationships on sexual lives during the lockdown period. In a study about male–female intimacy in a Chinese group, Feng et al. established that, in determining quality of intimacy, the independent contribution of family function was 48.8% [69]. The authors stated that in 40.5% of cases, family function was moderately impaired, and it affected intimacy between couples; participants with good family function had a higher degree of intimacy than those with severe family dysfunction [69]. Räuchle et al. underlined the role of psychological stress in generating conflict between partners and influencing sexual satisfaction [42]. Karagoz et al. described a correlation between the amount of time spent together during the pandemic and better sexual functioning scores in couples (men: *p* = 0.001, women: *p* = 0.006) [38]. According to Luetke et al., people experiencing frequent coronavirus-related conflict with their partner were significantly more likely to experience a decreased frequency of sexual behaviors compared to those not experiencing any such conflict [70]. Li G et al. noted that in 31% of their study group, new partnership conflicts emerged during the lockdown period [36]. Jacob et al. stated that being married or being in a domestic relationship is strongly correlated with frequency of sexual activity [65]. According to Li G et al., relationships were affected by factors such as sexual desire and satisfaction, relationship status, and place of residence during the pandemic [36]. These findings indicate far greater significance of the character of relationships on sexual functioning than our study. The reason for this might be the small number of married people in our study sample, which did not allow us to see such a correlation. This is probable, based on the study of Coombe et al., who observed that during lockdown, compared with 2019, people from their study group were more likely to report intercourse with a spouse (35.3% vs. 41.7%) and less likely to report intercourse with a girlfriend/boyfriend (45.1% vs. 41.8%), or casual intercourse (31.4% vs. 7.8%) [39].

Regarding sexual orientation, in this study, no significant correlation was found between sexual orientation and sexual functioning, both during the COVID-19 pandemic and before. Contradictory to these results, Batz et al. described being in a heterosexual relationship as associated with generally higher satisfaction with sexual life during the pandemic [56]. This correlation may have been missed in our study group because of an insufficient representation of LGBT+ people in committed relationships. Further research exploring this topic would be needed.

In this study, religiosity was found not to play a significant role in sexual functioning. The research by Fuchs et al. showed that religion in women has a statistically significant impact on levels of anxiety, which may affect sexual functioning in a negative way [60]. The differences presented might need further attention since, in this study, anxiety was not examined.

The COVID-19 pandemic is not the only widespread crisis that might have affected the overall health of the population, including sexual functioning. Different examples of such extreme situations include other epidemics, wars, or natural disasters.

The Ebola virus epidemic left a mark on the sexual life of the affected population. It is complicated to compare reports about the influence of the forementioned Ebola epidemic to the results of our study, because there is no research which compares the same aspects of sexual functioning as in our work. There have been, however, numerous negative occurrences regarding sexual functioning reported, which must have resulted in severe distress. The first of them was the permeation of the Ebola virus to the sperm, which forced a change in sexual behavior and the use of contraception [71,72]. Similarly to COVID-19, fears of intercourse infection appeared; however, in the case of the Ebola virus, this risk seemed substantially higher. In the population of Ghana, examined by Tenkorang, higher risk perception was associated with a higher probability of using barrier contraceptive methods [72]. Another problematic aspect was the decrease in the availability of reproductive health care. This occurred in part because, during the epidemic, such care was not considered a priority; in addition, it was also a result of the fear of potential viral infection during contact with health services. This resulted in a rise in adolescent pregnancy (because of the disrupted contraceptive care), as well as maternal and neonatal deaths [73,74,75,76]. In such circumstances, women’s health was impacted more negatively [77]. What is more, during the Ebola outbreaks, rape and sexual and gender-based violence increased, highlighting the vulnerabilities of women and girls to gender-based violence in humanitarian crises [78,79,80]. 

Similar tendencies have been described in relation to periods of armed conflict. According to Amnesty International, Actionaid International, and the United Nations, since the beginning of the war in Ukraine, there has been a significant rise in reports of sexual violence, including rape, sexual exploitation, and trafficking [81,82,83]. Such acts are often used as weapons of war to instill fear and exert control over populations. Many cases remain underreported due to stigma and fear of retribution. Women and girls are particularly vulnerable, facing increased risks of gender-based violence both within Ukraine and as refugees in neighboring countries [82]. Similarly to the case of the epidemic, the conflict has severely disrupted access to sexual and reproductive health services. Many healthcare facilities have been damaged, or are operating under constrained conditions, limiting access to essential services [81,82]. 

Another group which suffers because of armed conflicts are veterans, who may be affected by military sexual trauma or develop psychiatric conditions such as PTSD, which directly influences sexual functioning [84,85,86,87,88]. PTSD in veterans is a severe risk factor for developing sexual dysfunctions, such as disruptions to sexual arousal, sexual desire, and erectile function [85,86,87,88]. This is consistent with the results of a new study by Lazar et al., who examined the impact of war-related stressors on sexual well-being among Israeli civilians during the 2023 Israel–Hamas war. The study has shown that direct exposure to war stress is uniquely associated with sexual dysfunction, while media exposure and acute stress symptoms also significantly affect various aspects of sexual well-being [89].

When it comes to natural disasters, a study by Ebrahimian and Babaei assessed sexual dysfunction in married men affected by the Kermanshah earthquake. The results showed a 44.9% prevalence of sexual dysfunction, with significant differences in erectile function between affected and non-affected groups. The study highlighted the need for comprehensive attention to men’s sexual health in disaster recovery efforts [90].

Psychological traits and sexual beliefs may play a role as predisposing and maintaining factors for sexual dysfunction. According to Nobre et al., who examined the Portuguese population during the COVID-19 pandemic crisis in 2020, sexual functioning (measured with FSFI and IIEF) was negatively influenced by age, neuroticism, and (in females) age-related sexual beliefs (such as “As women age the pleasure they get from sex decreases”), while it was improved by high extraversion. High neuroticism and female age-related beliefs were also predictive factors for sexual distress. This study highlighted the fact that psychological traits and beliefs influence people’s response to environmental factors, such as the pandemic. This study may also explain the results of our study—the young age of our study population might have served as a protective factor against sexual dysfunction [91].

These data suggest that widespread crises may cause severe stress which results in health issues, including sexual dysfunction. This is especially notable in vulnerable groups such as veterans, women who are susceptible to violence, and people directly affected by natural disasters. In these cases, the negative impact of crises on sexual functioning seems to be much stronger than the influence of the COVID-19 pandemic, or even lockdown, on our study population, whose overall sexual functioning during the COVID-19 pandemic was not significantly different in comparison to the time before the pandemic. However, some threats may be of a similar nature to the risks of the pandemic, such as the adverse effects of the crisis on the mental health of the population, or worse access to some dimensions of healthcare.

## 5. Limitations

The limitations of this study are associated with the form of online survey employed. The use of such form had consequences, such as the subjective nature of the self-assessment of the respondents, self-report bias, and the risk of less thoughtfulness in answering the questionnaire in comparison to being questioned by an interviewer. The survey was distributed via social media, which may have influenced the study group by excluding individuals who do not use these platforms. However, the form of online survey probably enabled us to reach a larger group of participants.

Moreover, the assessment took place only once. In our questionnaire, sexual functioning prior to the pandemic was assessed retrospectively. This was due to the study design, which was established during the pandemic. What is more, the study lacked questions about occupation and having children, which makes it impossible to draw a conclusion about the influence of work–life balance on sexual functioning. 

Furthermore, because of the nature of the study design, the study group consisted of young people, so its results may not be relevant for the general population of all ages.

## 6. Directions for Future Research

More studies are needed on the topic of the impact of the pandemic on sexual health and mental well-being to fully understand the long-term impact of this worldwide health crisis. Further research is necessary to establish the influence of different forms of work and work–life balance on sexual functioning. It would also be helpful if future research included more age groups. To avoid limitations like ours regarding the research instruments, study protocols which involve interviewing participants in person and assessing them more than once, at different points in time, may be designed. This would enable researchers to describe changes in functioning across time more appropriately. 

## 7. Conclusions

The overall sexual functioning of young Polish individuals during the COVID-19 pandemic was not significantly different in comparison to the time before the pandemic. In comparison to the time before the COVID-19 pandemic, during the pandemic, women tended to function better in the aspects of pleasure and orgasm, whereas men tended to function worse in terms of erection and ejaculation. Significantly, more women than men suffered from overall sexual dysfunction both before and during the pandemic. The medians of the total CSFQ-14 scores for men and women were indicative of normal sexual functioning, whereas the specific subscales of the CSFQ-14 showed median scores in the dysfunction range regarding the time of pandemic and before it. This suggests that good overall sexual functioning does not exclude the possibility of dysfunction in some aspects of sexual life. Before the COVID-19 pandemic, remote work seemed to positively influence the sexual functioning of women, especially in terms of desire and frequency, and orgasm. During the COVID-19 pandemic, remote work influenced the sexual functioning of women negatively, especially in the aspects of desire and interest, and arousal.

## Figures and Tables

**Table 1:** Study group characteristics.

Feature	Variant of the Feature	N (%)		
		In the Whole Group	In the Male Group	In the Female Group
Sex	Male	123 (22.74%)	-	-
	Female	418 (77.26%)	-	-
Place of residence	Up to 10,000 residents	132 (24.40%)	18 (14.63%)	114 (27.27%)
	10,000–50,000 residents	98 (18.11%)	18 (14.63%)	80 (19.14%)
	50,000–100,000 residents	66 (12.20%)	21 (17.07%)	45 (10.77%)
	100,000–250,000 residents	75 (13.86%)	16 (13.01%)	59 (14.11%)
	Over 250,000 residents	170 (31.42%)	50 (40.65%)	120 (28.71%)
Religiosity	Believers practicing religion	111 (20.52%)	21 (17.07%)	90 (21.53%)
	Believers not practicing religion	223 (41.22%)	41 (33.33%)	182 (43.54%)
	Atheists	207 (38.26%)	61 (49.60%)	146 (34.93%)
Relationship status	Single	100 (18.49%)	36 (29.27%)	64 (15.31%)
	In an informal relationship	385 (71.16%)	80 (65.04%)	305 (72.97%)
	Engaged	46 (8.50%)	6 (4.88%)	40 (9.57%)
	Married	10 (1.85%)	1 (0.81%)	9 (2.15%)
Sexual orientation	Heterosexual	436 (80.59%)	101 (82.11%)	335 (80.14%)
	Homosexual	19 (3.51%)	8 (6.51%)	11 (2.63%)
	Bisexual/pansexual	78 (14.42%)	14 (11.38%)	64 (15.31%)
	Other	8 (1.48%)	0 (0%)	8 (1.92%)
Being quarantined or self-isolation because of COVID-19	Yes	187 (34.57%)	49 (39.84%)	138 (33.01%)
	No	354 (65.43%)	74 (60.16%)	280 (66.99%)
Cohabitation with a partner before the pandemic	Yes	82 (15.16%)	12 (9.76%)	70 (16.75%)
	No	459 (84.84%)	111 (90.24%)	348 (83.25%)
Cohabitation with a partner during lockdown period	Yes	122 (22.55%)	21 (17.07%)	101 (24.16%)
	No	419 (77.45%)	102 (82.93%)	317 (75.84%)
Remote work before the pandemic	Yes	50 (9.24%)	18 (14.63%)	32 (7.66%)
	No	491 (90.76%)	105 (85.37%)	386 (92.34%)
Remote work during lockdown period	Yes	73 (13.49%)	24 (19.51%)	49 (11.72%)
	No	468 (86.51%)	99 (80.49%)	369 (88.28%)
Remote university studies before the pandemic	Yes	157 (29.02%)	36 (29.27%)	121 (28.95%)
	No	384 (70.98%)	87 (70.73%)	297 (71.05%)
Remote university studies during lockdown period	Yes	376 (69.50%)	89 (72.36%)	287 (68.66%)
	No	165 (30.50%)	34 (27.64%)	131 (31.34%)
Age (median)	-	21	22	21

**Table 2:** Total CSFQ-14 scores of both groups (male and female).

Sex	Time Period	Median	Mean	Percentage of Scores Indicative of Normal Function	Percentage of Scores Indicative of Sexual Dysfunction
Male	Before the pandemic	60	59.41	98.37%	1.63%
	During the pandemic	60	58.88	95.12%	4.88%
Female	Before the pandemic	52.5	51.07	85.41%	14.59%
	During the pandemic	53	52.20	87.80%	12.20%

**Table 3:** Total CSFQ-14 scores of women in subgroup differentiated by remote work.

Feature	Variant of the Feature	Before the COVID-19 Pandemic		During the COVID-19 Pandemic	
		Median CSFQ-14 Score	*p*	Median CSFQ-14 Score	*p*
Remote work	Yes	54	*p* > 0.05	50	*p* = 0.004
	No	52		54	

**Table 4:** Percentages of people with a total CSFQ-14 score indicative of normal sexual function in subgroup differentiated from the whole study group by sex.

Feature	Variant of the Feature	Percentage of People with Total CSFQ-14 Score Indicative of Normal Sexual Functioning			
		Before the COVID-19 Pandemic	*p*	During the COVID-19 Pandemic	*p*
Sex	Male	98.37%	*p* = 0.0002	95.12%	*p* = 0.02
	Female	85.41%		87.80%	

**Table 5:** Percentages of people with a total CSFQ-14 score indicative of normal sexual function in subgroup differentiated by working remotely.

Group	Remote Work	Percentage of People with Total CSFQ-14 Score Indicative of Normal Sexual Functioning			
		Before the COVID-19 Pandemic	*p*	During the COVID-19 Pandemic	*p*
All	Yes	98.00%	*p* = 0.045	83.56%	*p* > 0.05
	No	87.37%		90.38%	
Male	Yes	100.00%	*p* > 0.05	91.67%	*p* > 0.05
	No	98.10%		95.96%	
Female	Yes	96.88%	*p* = 0.004	79.59%	*p* > 0.05
	No	84.46%		88.89%	

**Table 6:** Median and mean scores obtained by male and female participants in different CSFQ-14 subscales in relation to the periods before and during the COVID-19 pandemic.

Sex	Subscale	Period	Median	Mean	*p* (Wilcoxon’s Test)
Male	Pleasure	Before the COVID-19 pandemic	4	3.84	*p* > 0.05
		During the COVID-19 pandemic	4	3.84	
	Desire/frequency	Before the COVID-19 pandemic	8	8.05	*p* > 0.05
		During the COVID-19 pandemic	8	7.89	
	Desire/interest	Before the COVID-19 pandemic	11	11.00	*p* > 0.05
		During the COVID-19 pandemic	11	11.18	
	Arousal/erection	Before the COVID-19 pandemic	14	13.85	*p* = 0.003
		During the COVID-19 pandemic	14	13.41	
	Orgasm/ejaculation	Before the COVID-19 pandemic	14	13.27	*p* = 0.022
		During the COVID-19 pandemic	13	13.07	
Female	Pleasure	Before the COVID-19 pandemic	4	3.75	*p* = 0.019
		During the COVID-19 pandemic	4	3.96	
	Desire/frequency	Before the COVID-19 pandemic	7	7.04	*p* > 0.05
		During the COVID-19 pandemic	7	7.17	
	Desire/interest	Before the COVID-19 pandemic	10	9.66	*p* > 0.05
		During the COVID-19 pandemic	10	9.68	
	Arousal/excitement	Before the COVID-19 pandemic	12	11.25	*p* > 0.05
		During the COVID-19 pandemic	12	11.51	
	Orgasm/completion	Before the COVID-19 pandemic	11	10.72	*p* = 0.005
		During the COVID-19 pandemic	12	11.25	

**Table 7:** Percentage of women with scores of CSFQ-14 pleasure subscale indicative of sexual dysfunction before and during the COVID-19 pandemic.

Group	Scale	Period	Percentage of People with Score Indicating Sexual Dysfunction *	*p*
Female	Pleasure	Before the COVID-19 pandemic	73.44%	*p* < 0.00001
		During the COVID-19 pandemic	59.57%	

* Cut-off point for pleasure dysfunction: score ≤ 4.0.

**Table 8:** Percentages of men and women showing sexual dysfunction in different CSFQ-14 subscales before and during the COVID-19 pandemic.

Subscale	Sex	Percentage of People with Score Indicating Sexual Dysfunction			
		Before the COVID-19 Pandemic		During the COVID-19 Pandemic	
		%	*p*	%	*p*
Desire/frequency	Male	64.23%	*p* < 0.0001	50.41%	*p* < 0.0001
	Female	34.21%		43.06%	
Desire/interest	Male	58.54%	*p* = 0.01	50.41%	*p* > 0.05
	Female	45.45%		43.06%	
Arousal/erection Arousal/excitement	Male	32.52%	*p* < 0.0001	39.84%	*p* < 0.0001
	Female	67.22%		62.44%	

**Table 9:** Median scores of CSFQ-14 subscales in subgroup of women differentiated by undertaking remote work before and during the pandemic.

Subscale	Remote Work	Median CSFQ-14-F Subscales Scores			
		Before the COVID-19 Pandemic	*p*	During the COVID-19 Pandemic	*p*
Pleasure	Yes	4	*p* > 0.05	4	*p* > 0.05
	No	4		4	
Desire/frequency	Yes	8	*p* = 0.01	7	*p* > 0.05
	No	7		8	
Desire/interest	Yes	10	*p* > 0.05	9	*p* = 0.01
	No	10		10	
Arousal/excitement	Yes	12	*p* > 0.05	11	*p* = 0.003
	No	12		12	
Orgasm/completion	Yes	12.5	*p* = 0.03	11	*p* > 0.05
	No	11		12	

## Data Availability

Patients data are available from H.P. and A.W.
